# The Frequency of Semen Abnormalities Among Jordanian Population: A Retrospective Single-Center Study of 1182 Cases

**DOI:** 10.3390/diseases14060213

**Published:** 2026-06-12

**Authors:** Fatina W. Dahadhah, Mohanad Odeh, Adnan A. Dahadha, Heba A. Ali, Eman Hussein Alshdaifat, Emad Freihat, Manal Issam Abu Alarjah, Mazhar Salim Al Zoubi

**Affiliations:** 1 Department of Basic Dental Sciences, Faculty of Dentistry, The Hashemite University, Zarqa 13115, Jordan; 2 Department of Clinical Pharmacy and Pharmacy Practice, Faculty of Pharmaceutical Sciences, The Hashemite University, Zarqa 13115, Jordan; 3 Department of Biotechnology and Genetic Engineering, Faculty of Science, Philadelphia University, Amman P.O. Box 19392, Jordan; 4 Department of Obstetrics and Gynecology, Faculty of Medicine, Yarmouk University, Irbid 21163, Jordan; 5 Reproductive Endocrinology and IVF Unit, King Hussein Medical Center, Amman 11733, Jordan; 6 Department of Basic Medical Sciences, Faculty of Medicine, Yarmouk University, Irbid 21163, Jordan

**Keywords:** sperm parameters, male infertility, semen abnormalities

## Abstract

Background/Objectives: Male infertility is a significant global public health concern, with sperm abnormalities contributing to nearly half of all cases. This retrospective study addressed a critical gap by analyzing 1182 semen records from subfertile Jordanian men at the IVF unit. Methods: We quantified the prevalence of specific abnormalities, oligozoospermia, asthenozoospermia, teratozoospermia, and azoospermia, and investigated the interrelationships between sperm count, motility, and morphology. Results: Oligozoospermia was the most prevalent single abnormality (29%), while normozoospermia was found in 28% of cases. Combined defects were frequent; oligoasthenoteratozoospermia, with low count, poor motility, and abnormal morphology, was identified in 17% of cases, highlighting the multifactorial nature of subfertility. Correlation analysis showed significant interdependencies. A strong positive correlation was found between normal morphology and progressive motility (r = 0.484, *p* < 0.0001), suggesting structural integrity is closely linked to movement. Sperm count showed moderate positive correlations with normal morphology (r = 0.508, *p* < 0.0001) and non-progressive motility (r = 0.522, *p* < 0.0001). Abnormal morphology exhibited the strongest positive correlation with immotile sperm (r = 0.559, *p* < 0.0001), consistent with asthenoteratozoospermia. Conclusions: These findings suggest semen issues in Jordanian men include multiple parameters simultaneously. Comprehensive semen analysis is essential for accurate fertility assessment, as focusing on a single parameter like sperm count may overlook critical factors contributing to infertility.

## 1. Introduction

Male factor infertility presents in more than 40% of couples seeking assisted reproduction treatment [1]. Conventional semen analysis remains the only routine test for diagnosing this condition, even though it is known that such descriptive assessments cannot provide a clear picture of the underlying problem [2]. Increasing shards of evidence are available suggesting that male infertility has a genetic link [3].

According to the World Health Organization, male infertility can be diagnosed through a traditional semen analysis. This analysis evaluates various factors, including semen volume and liquefaction, anti-sperm antibodies, sperm count, morphology, and motility [4]. Sperm motility is essential for successful fertilization [5]. It has been found that around 75% of infertile men have either oligozoospermia, characterized by a reduced sperm count, or asthenozoospermia, where most of the sperm are unable to move [6,7].

The typical semen parameters that indicate normal fertility encompass the following criteria: complete liquefaction within 60 min, a volume of 1.5 mL, a grey-white color, a pH level above 7.1, a sperm concentration exceeding 15 million sperm per mL, a motility percentage above 40%, and at least 4% of sperm with normal morphology. It is important to note that male infertility typically presents with lower sperm parameters [8].

Infertility cases are diagnosed based on three critical categories: sperm count, morphology, and motility.

### 1.1. Asthenozoospermia

Sperm motility refers to the sperm’s ability to move and is classified into four types: progressive (sperm moving rapidly in a straight line), non-progressive (sperm moving without forward progression, quick but sluggish), local (sperm shaking or vibrating on the same spot), and immotile (sperm showing no movement). Asthenozoospermia is diagnosed when fewer than 40% of sperm are motile (the combined progressive and non-progressive motility is below 40%) [8,9].

### 1.2. Oligozoospermia

Oligozoospermia refers to a condition characterized by sperm concentrations below 15 million sperm per milliliter of ejaculate. This abnormality is further classified into three categories based on severity. In cases of oligozoospermia, the sperm count ranges between 100,000 and 15 million/mL. Severe oligozoospermia is diagnosed when the sperm concentration falls below 100,000/mL, indicating a more profound reduction in spermatogenesis. The most extreme form, azoospermia, is identified when there is a complete absence of sperm in one milliliter of seminal fluid. These classifications are essential for the clinical evaluation and management of male infertility [8,10].

### 1.3. Teratozoospermia

Sperm morphology pertains to the shape and structure of the sperm cell. Any abnormalities or defects in sperm morphology can significantly contribute to male infertility, as outlined by the World Health Organization in 2010 [8].

There are many factors associated with male infertility, including age, stress, cigarette smoking, and genetic factors. A study carried out in Jordan revealed that infertile couples in Jordan have psychological and social problems. It highlighted concerning levels of moderate-to-severe depression among these couples, emphasizing the need for comprehensive clinical assessment and ongoing support [11].

Moreover, genetic disorders play a significant role in about 15–30% of male factor infertility, often resulting from sperm abnormalities, such as asthenozoospermia. This condition has been linked to mutations in mitochondrial DNA (mtDNA). One common mutation in sperm is the 4977 bp human mtDNA deletion, which results in the loss of approximately 33% of the mitochondrial genome. A recent study in Jordan investigated the presence of a 4977 bp mtDNA deletion in infertile men with asthenozoospermia, and the findings revealed a strong association between the deletion and asthenozoospermia in the Jordanian population [12].

About 30% of men visiting infertility clinics were diagnosed with oligozoospermia, oligoasthenoteratozoospermia (OAT), or azoospermia of an unknown etiology. In infertile men, sperm motility is usually the foremost cause of infertility, and abnormal sperm motility could be linked to multiple deletions in the mitochondrial DNA [12]. ATPs are the fuel to move sperm and help them reach the fallopian tubes, leading to fertilization [13].

Semen analysis remains the most valuable and essential test for identifying male infertility. With a sensitivity of 89.6%, it can detect genuine infertility problems in 9 out of 10 men [14,15].

Previous research in Jordan has primarily focused on specific genetic and clinical aspects of male infertility, particularly among azoospermic and oligozoospermic men attending infertility and IVF centers [12,16,17,18]. While these studies have offered important insights into the roles of genetic and lifestyle-related factors in male infertility, there is a lack of comprehensive epidemiological analyses assessing the frequency, coexistence, and interrelationships of semen abnormalities in large populations of infertile Jordanian men.

Moreover, Male infertility has significant social and psychological consequences in the Middle East; however, epidemiological trends are insufficiently reported. By analyzing a cohort of 1182 cases, our study addresses this regional gap by mapping the distribution of parameters, including oligozoospermia, asthenozoospermia, and teratozoospermia, thereby informing local reproductive health strategies and fertility management frameworks.

Therefore, we aim to determine the frequency of sperm abnormalities that cause male infertility in Jordanian males attending the in vitro *fertilization* unit of Royal Jordanian Medical Services Hospitals, including asthenozoospermia, oligozoospermia, teratozoospermia, and azoospermia, and to assess the frequency of sperm count, motility, and morphology to understand the variations in their prevalence in our region.

## 2. Methods

### 2.1. Study Design and Setting

This retrospective study was conducted at the in vitro *fertilization* unit of Royal Medical Services Hospitals, following ethics approval from the Institutional Ethical Committee.

### 2.2. Study Population and Data Collection

The subjects were not directly involved in this study; it included an archival review of 1182 seminal analysis results collected between 1 January 2023, and 31 December 2023. All data were obtained from the patients’ medical records, including parameters such as volume, pH, viscosity, liquefaction time, agglutination, motility, count, and morphology, in accordance with the World Health Organization guidelines 2021 [8].

To ensure patient privacy, the patients’ names and personal information were not collected. All data were treated confidentially and used only for the purposes of this study.

The analysis was conducted using a correlation matrix of quantitative data for semen parameters, including concentration, morphology, and motility, and the frequency of each variable was identified.

### 2.3. Inclusion and Exclusion Criteria

Inclusion criteria required a documented history of infertility, defined as failure to conceive after at least 12 months of regular, unprotected sexual intercourse. All semen analyses from subfertile men attending the IVF unit between 1 January 2023, and 31 December 2023, were considered. Records were excluded if incomplete or lacking semen analysis results. Duplicate or repeated semen samples from the same patients were excluded when identifiable to avoid overrepresentation.

### 2.4. Statistical Analysis

Data were screened for completeness, consistency, missing values, outliers, and biologically implausible values before analysis. Continuous variables were summarized using mean, median, standard deviation, range, and interquartile range, while categorical semen diagnoses were reported as frequencies and percentages.

The normality of continuous variables was assessed using the Shapiro–Wilk test, supported by inspection of descriptive statistics, histograms, and Q–Q plots. Because several semen parameters showed non-normal distributions, skewed values, and wide ranges, associations between quantitative variables were examined using Spearman’s rank correlation coefficient. Correlation strength was interpreted according to the absolute value of the coefficient as follows: negligible (<0.10), weak (0.10–0.29), moderate (0.30–0.49), strong (0.50–0.69), and very strong (≥0.70). Correlation results were presented as Spearman’s rho coefficients with corresponding *p*-values. All tests were two-sided, and statistical significance was set at *p* < 0.05. Given the large sample size, interpretation emphasized both statistical significance and the magnitude and direction of associations. All analyses were performed using IBM SPSS Statistics, version 27.0.

### 2.5. Use of Generative AI for Language Support

During the preparation of this manuscript, ChatGPT version 5.5 (OpenAI) and Grammarly Pro were utilized as language-support tools to assist with editing, clarification of wording, and the improvement of coherence and academic writing style. No generative AI or AI-assisted tools were used for data collection, data analysis, interpretation of results, or the generation of scientific content. All references cited in this manuscript were independently selected, verified, and checked by the authors; no references were generated using AI tools.

## 3. Results

### 3.1. Demographic and Semen Characteristics of the Study Population

The mean age of participants was 33.95 years (SD = 6.51), with an interquartile range (IQR) of 8.0 years. The average abstinence period before sample collection was 4.14 days (SD = 1.69), with an IQR of 2.0 days. The mean ejaculate volume was 3.12 mL (SD = 2.00; IQR = 2.0), and the mean sperm count per ejaculate was 29.65 million (SD = 20.77), with an IQR of 31.5 million. Normal sperm morphology had a mean of 7.77% (SD = 7.42; IQR = 10.0), reflecting notable variability across samples (Table 1).

### 3.2. Frequency of Semen Abnormalities

The most common diagnosis was oligozoospermia, accounting for 345 cases (29%), followed by normozoospermia with 333 cases (28%). Oligoasthenoteratozoospermia was identified in 201 cases (17%), while Oligoteratozoospermia represented 94 cases (8.0%) (Table 2).

These findings suggest that the sample population shows a moderately consistent age and abstinence range, supporting controlled sampling conditions. However, the considerable variation in sperm count and morphology indicates a heterogeneous fertility profile among participants. The low average percentage of normal morphology is consistent with clinical observations that only a small proportion of sperm typically meet strict morphological criteria. Together, these descriptive statistics provide a foundational overview of semen quality within the studied group, informing further analysis of relational and clinical outcomes.

### 3.3. Correlation Analysis of Semen Parameters

Table 3 shows the correlation analysis of semen parameters, which revealed several statistically significant relationships, highlighting key interdependencies among indicators of male reproductive function. The strongest observed correlation was between abnormal morphology and immotile sperm (r = 0.559, *p* < 0.0001), demonstrating a significant association between morphologically abnormal sperm and reduced motility. A significant positive relationship has been observed between sperm count and non-progressive motility (r = 0.522, *p* < 0.0001). A comparable correlation strength was found between sperm count and normal morphology (r = 0.508, *p* < 0.0001).

Further significant positive associations were observed between normal morphology and non-progressive motility (r = 0.488, *p* < 0.0001) and between normal morphology and progressive motility (r = 0.484, *p* < 0.0001). Additionally, abnormal morphology and local motility showed a moderate positive correlation (r = 0.292, *p* < 0.0001). In contrast, normal morphology and immotile sperm were negatively correlated (r = −0.291, *p* < 0.0001), reinforcing that better morphology corresponds with reduced immotility.

Sperm count was also significantly associated with local motility (*r* = 0.448, *p* < 0.0001) and progressive motility (*r* = 0.339, *p* < 0.0001) and negatively associated with immotile sperm (*r* = −0.290, *p* < 0.0001). Likewise, normal morphology and local motility were positively associated (*r* = 0.336, *p* < 0.0001), reflecting the integrated nature of sperm form and function.

Moderate correlations were also noted between count and abnormal morphology (*r* = 0.195, *p* < 0.0001), and between volume and non-progressive motility (*r* = 0.131, *p* < 0.0001), suggesting minor yet meaningful links between ejaculate volume and sperm motility. Weaker, yet statistically significant, relationships were observed between abstinence and count (*r* = 0.074, *p* = 0.0108), abstinence and volume (*r* = 0.068, *p* = 0.0189), and abnormal and normal morphology (*r* = −0.067, *p* = 0.0223).

Finally, weak but significant positive correlations were observed between abstinence duration and non-progressive motility (r = 0.031, *p* = 0.0283), and no significant correlation was observed between normal morphology and volume (r = 0.033, *p* = 0.2593).

## 4. Discussion

Infertility affects approximately 10–15% of the global population, amounting to an estimated 50–80 million couples who experience difficulties conceiving [19,20]. Male factor infertility plays a substantial role in this burden, contributing to roughly 40–50% of cases and affecting about 7% of all men [14]. It is clinically defined as the inability to achieve pregnancy after 12 months of regular unprotected intercourse [21], with a man being diagnosed as the contributing factor when his sperm parameters fall below the normal thresholds established by the World Health Organization [22]. Such subnormal parameters commonly manifest as low sperm concentration (oligozoospermia), reduced sperm motility (asthenozoospermia), or abnormal sperm morphology (teratozoospermia). Semen analysis remains the most valuable and essential test for identifying male infertility. With a sensitivity of 89.6%, it can detect genuine infertility problems in 9 out of 10 men [14,15].

Our study provides a comprehensive examination of semen parameters among Jordanian men, addressing a notable lack of prior research on infertility in this population. Three major parameters were tested, including oligozoospermia, Asthenozoospermia, and teratozoospermia, and comprehensively analyzed in the current study. In our cohort (*N* = 1182), oligozoospermia was detected in 29% of participants, while only 6% had azoospermia. Asthenozoospermia and teratozoospermia were comparatively infrequent in their pure forms (approximately 2% and 1% of cases, respectively). By combining data, oligozoospermia cases showed the highest frequency among Jordanian males, followed by azoospermia, and the least frequent were asthenozoospermia and teratozoospermia, respectively. This distribution suggests that many infertile men experience concurrent impairments across multiple sperm parameters, rather than isolated deficiencies.

Comparative data from a tertiary hospital in Eastern India reported a similar 24% rate among infertile men [23]. Regional differences are seen across India; a multi-city study of 16,714 infertile men found a higher 51% in Kurnool, while other areas had much lower rates [24]. These differences reflect local population and healthcare factors. Our 29% rate is below the highest regional numbers but matches more typical rates seen in India, highlighting the need to consider local context when interpreting findings.

When examining azoospermia, the absence of sperm in the ejaculate, our study found a frequency of 6%. According to the American Society for Reproductive Medicine (ASRM), azoospermia is reported in approximately 10% to 15% of men presenting with infertility. This variation underscores the importance of understanding local epidemiological patterns when assessing male infertility profiles [25].

The relatively high frequency of OAT syndrome observed in our cohort highlights the importance of comprehensive semen evaluation. OAT is recognized as a particularly consequential disorder due to its triple impact on sperm count, motility, and morphology [26]. A recent study from Central India reported an even higher OAT prevalence of 34% [27], exceeding the rates of other individual semen abnormalities in that sample. While our OAT rate is lower, both findings affirm that OAT is a common and clinically important condition. They also illustrate the necessity of evaluating all three sperm parameters together; if clinicians focus on only one aspect, they might overlook a significant subset of patients who have moderate deficiencies across multiple domains rather than an extreme defect in a single parameter.

The relatively high frequency of OAT syndrome identified in this cohort has significant clinical implications. Combined abnormalities in sperm concentration, motility, and morphology are commonly linked to more severe forms of male infertility and may indicate multifactorial disruptions in spermatogenesis that require comprehensive evaluation, including hormonal, genetic, and lifestyle assessments [28,29,30]. Previous studies have demonstrated that severe male factor infertility, particularly OAT syndrome, often necessitates the use of assisted reproductive techniques such as intracytoplasmic sperm injection (ICSI) to enhance fertilization outcomes [31,32]. Furthermore, genetic counseling and screening for chromosomal abnormalities or Y-chromosome microdeletions may be indicated in severe cases prior to assisted reproduction [33,34].

The high frequency of male infertility in Jordan may be related to multiple factors. Psychosocial health is an important factor, as stress between couples showed a significant impact on infertility rates in Jordan, as reported in a recent study, which emphasized the necessity for specific psychological therapies that are currently lacking in public healthcare practices [35].

The second factor concerns couples’ lifestyles. In Jordan, almost 39% of men smoked more than 5 cigarettes per day, and almost 22% engaged in regular sporting activity. High cigarette consumption and low physical activity elevate the risk of male infertility among the Jordanian population [36].

The most essential and common factor is the population’s genetic makeup. The Jordan population showed varied genetic causes, as follows: first, the androgen receptor CAG repeat length in infertile males. There is also a significant association between AR-CAG length polymorphism and oligozoospermia and teratozoospermia, but not in azoospermic cases, in which abnormal cases have longer repeats of AR-CAG polymorphism [12].

The MTHFR 677TT genotype is also considered as one of the essential genetic factors that were detected more in infertile Jordanian males compared to normal males [16] and increases the risk of males being infertile. Regarding the genetic contribution, Y chromosome microdeletion is one of the leading causes of male infertility in Jordan [17,18].

Beyond prevalence rates and risk factors, our study also examined how various semen parameters correlate with one another, which offers insight into the underlying biology of sperm quality. The correlation matrix of our data revealed several statistically significant findings. Most strikingly, we observed a strong positive correlation between normal sperm morphology and progressive motility, quantified as Spearman’s r = 0.54 (*p* < 0.0001). This aligns closely with a seminal study by Parinaud et al. [37], which identified a similarly robust correlation in human semen samples: r = 0.539, *p* < 0.0001. This strong consistency between our data and established findings reinforces the conclusion that better morphological integrity is tightly and significantly linked to forward sperm movement. Such a statistically validated relationship underlines the integrated nature of structural and functional parameters in semen analysis, solidifying morphology’s critical role in fertility assessments [37].

Moreover, we observed a strong positive correlation between sperm count and the percentage of morphologically normal sperm, quantified as r = 0.508 (*p* < 0.001). This finding reflects a common pattern reported in a study that found a moderate positive correlation between motile sperm concentration and morphology scores (strict criteria: r = 0.45; non-strict criteria: r = 0.51). These results mirror our observations, reinforcing the idea that higher sperm counts tend to coincide with improved morphological integrity [38].

Importantly, Coutton and his colleagues provide mechanistic support: they showed that biallelic mutations in CFAP43 and CFAP44 cause multiple morphological abnormalities of sperm flagella (MMAF) associated with complete sperm immotility [39].

The intermediate motility categories provided additional insights. Non-progressive motility (non-progressive motility) showed the strongest correlation with sperm count (r = 0.522, *p* < 0.0001), suggesting that while sperm quantity may promote movement, it does not necessarily ensure directional progression. Local motility exhibited moderate associations with abnormal morphology, further supporting the relationship between sperm structural abnormalities and impaired motility.

Interestingly, we found that the abstinence period showed only weak associations with semen parameters, the most notable being with sperm count (r = 0.074, *p* = 0.0108). This modest relationship supports current guidelines recommending 2–7 days of abstinence for optimal semen analysis results [8], while suggesting that abstinence duration has a limited impact on more functional parameters like motility.

Collectively, these findings emphasize the importance of integrated semen assessment in male infertility evaluation and support the need for individualized diagnostic and therapeutic approaches for patients with combined semen abnormalities.

## 5. Limitations

The retrospective, single-center design limits the ability to establish direct causal relationships and relies primarily on existing archival records. This reliance prevented the evaluation of broader clinical, lifestyle, and anthropometric variables, including smoking status, body weight, and occupational exposures, which may influence male fertility parameters. Additionally, due to limitations in archived clinical documentation, detailed clinical histories, medication use, and prior infertility treatments were not consistently available and could not be systematically controlled. Future prospective multicenter studies incorporating hormonal, genetic, lifestyle, and reproductive outcome data are needed to better characterize male infertility patterns in Jordanian populations.

## 6. Conclusions

In summary, our findings highlight the multifactorial nature of male infertility among Jordanian men and demonstrate that abnormalities in sperm count, motility, and morphology are often interrelated rather than occurring independently. These findings reinforce the clinical value of a comprehensive semen analysis: evaluating all parameters together provides a more complete assessment of male fertility potential than isolated tests. Finally, we recommend that future longitudinal studies track semen quality in Jordanian men over time to detect changing prevalence patterns and emerging lifestyle or genetic risk factors. Such long-term monitoring will be crucial for guiding public health strategies and improving the management of male infertility in this population.

## Figures and Tables

**Table 1:** Descriptive statistics of studied parameters for all subjects (N = 1182).

Parameter	Mean	Median	Std	Range	IQR
**Age**	33.95	33.00	6.51	70.00	8.00
**Abstinence**	4.14	4.00	1.69	40.00	2.00
**Volume**	3.13	3.00	2.00	40.00	2.00
**Count**	29.66	29.00	20.78	100.00	31.75
**Normal Morphology**	7.76	7.00	7.42	100.00	10.00
**Abnormal Morphology**	86.57	92.00	22.39	100.00	10.00
**Progressive Motility**	1.69	0.00	3.81	55.00	2.00
**Non-progressive Motility**	9.88	8.00	8.96	70.00	17.00
**Local Motility**	34.21	35.00	20.92	90.00	28.00
**Immotile Sperm**	48.78	45.00	25.93	100.00	33.00

**Table 2:** The frequency of studied parameters for all subjects (N = 1182).

Category	Number	Frequency (%)
**Oligozoospermia**	345	29%
**Normozoospermia**	333	28%
**Oligoteratoasthenozoospermia**	207	18%
**Oligoteratozoospermia**	94	8%
**Oligoasthenozoospermia**	81	7%
**Azoospermia**	73	6%
**Asthenozoospermia**	27	2%
**Teratozoospermia**	12	1%
**Teratoasthenozoospermia**	10	1%

**Table 3:** Correlation matrix of quantitative data.

	Age	Abstinence	Volume	Count	Normal_ Morphology	Abnormal_ Morphology	Progressive Motility	Non-Progressive Motility	Local Motility	Immotile Sperm
**Age**	r = 1.000,	r = −0.049, *p* = 0.0916	r = 0.001, *p* = 0.9756	r = −0.027, *p* = 0.362	r = −0.040, *p* = 0.1709	r = −0.047, *p* = 0.1111	r = −0.008, *p* = 0.7734	r = −0.057, *p* = 0.0502	r = −0.047, *p* = 0.1072	r = −0.000, *p* = 0.9872
**Abstinence**	r = −0.049, *p* = 0.0916	r = 1.000,	r = 0.068, *p* = 0.0189	r = 0.074, *p* = 0.0108	r = 0.008, *p* = 0.7934	r = 0.005, *p* = 0.8625	r = −0.014, *p* = 0.63	r = 0.031, *p* = 0.283	r = −0.003, *p* = 0.9062	r = −0.006, *p* = 0.8337
**Volume**	r = 0.001, *p* = 0.9756	r = 0.068, *p* = 0.0189	r = 1.000	r = −0.004, *p* = 0.895	r = 0.033, *p* = 0.2593	r = 0.057, *p* = 0.0526	r = 0.025, *p* = 0.3824	r = 0.131, *p* < 0.001	r = 0.009, *p* = 0.7659	r = 0.002, *p* = 0.9571
**Count**	r = −0.027, *p* = 0.362	r = 0.074, *p* = 0.0108	r = −0.004, *p* = 0.895	r = 1.000	r = 0.508, *p* < 0.001	r = 0.195, *p* < 0.001	r = 0.339, *p* < 0.001	r = 0.522, *p* < 0.001	r = 0.448, *p* < 0.001	r = −0.290, *p* < 0.001
**Normal Morphology**	r = −0.040, *p* = 0.1709	r = 0.008, *p* = 0.7934	r = 0.033, *p* = 0.2593	r = 0.508, *p* < 0.001	r = 1.000	r = −0.067, *p* = 0.012	r = 0.484, *p* < 0.001	r = 0.488, *p* < 0.001	r = 0.336, *p* < 0.001	r = −0.291, *p* < 0.001
**Abnormal_ Morphology**	r = −0.047, *p* = 0.1111	r = 0.005, *p* = 0.8625	r = 0.057, *p* = 0.0526	r = 0.195, *p* < 0.001	r = −0.067, *p* = 0.0223	r = 1.000	r = −0.048, *p* = 0.0983	r = 0.119, *p* < 0.001	r = 0.292, *p* < 0.001	r = 0.559, *p* < 0.001
**Progressive motility**	r = −0.008, *p* = 0.7734	r = −0.014, *p* = 0.63	r = 0.025, *p* = 0.3824	r = 0.339, *p* < 0.001	r = 0.484, *p* < 0.001	r = −0.048, *p* = 0.0983	r = 1.000	r = 0.401, *p* < 0.001	r = 0.106, *p* = 0.0003	r = −0.278, *p* < 0.001
**Non-progressive motility**	r = −0.057, *p* = 0.0502	r = 0.031, *p* = 0.0283	r = 0.131, *p* < 0.001	r = 0.522, *p* < 0.001	r = 0.488, *p* < 0.001	r = 0.119, *p* < 0.001	r = 0.401, *p* < 0.001	r = 1.000,	r = 0.218, *p* < 0.001	r = −0.348, *p* < 0.001
**Local motility**	r = −0.047, *p* = 0.1072	r = −0.003, *p* = 0.9062	r = 0.009, *p* = 0.7659	r = 0.448, *p* < 0.001	r = 0.336, *p* < 0.001	r = 0.292, *p* < 0.001	r = 0.106, *p* = 0.0003	r = 0.218, *p* < 0.001	r = 1.000	r = −0.560, *p* < 0.001
**Immotile sperm**	r = −0.000, *p* = 0.9872	r = −0.006, *p* = 0.8337	r = 0.002, *p* = 0.9571	r = −0.290, *p* < 0.001	r = −0.291, *p* < 0.001	r = 0.559, *p* < 0.001	r = −0.278, *p* < 0.001	r = −0.348, *p* < 0.001	r = −0.560, *p* < 0.001	r = 1.000

## Data Availability

Data are available upon request owing to privacy/ethical restrictions.
